# A Rare Clinical Conundrum: Cervical Ectopic Pregnancy in the Presence of a Large Cervical Fibroid

**DOI:** 10.7759/cureus.90591

**Published:** 2025-08-20

**Authors:** Vineeta Singh, Divya M Balachandran, Amiy Arnav, Habib Md R Karim, Vikash Bansal

**Affiliations:** 1 Obstetrics and Gynecology, All India Institute of Medical Sciences, Deoghar, IND; 2 Surgical Oncology, All India Institute of Medical Sciences, Deoghar, IND; 3 Anesthesiology, Critical Care, and Pain Medicine, All India Institute of Medical Sciences, Assam, IND; 4 Anesthesia, Critical Care, and Pain Medicine, All India Institute of Medical Sciences, Deoghar, IND

**Keywords:** cervical fibroid, cervical pregnancy, heavy menstrual bleeding, hysterectomy, pregnancy

## Abstract

Cervical ectopic pregnancy is a rare and potentially life-threatening condition, often presenting with irregular vaginal bleeding and a palpable cervical mass. The coexistence of cervical fibroids further complicates the diagnosis and management.

A 29-year-old woman, P_4_L_4_, presented with a history of three months of irregular and heavy vaginal bleeding. She also reported recurrent urinary tract infections and difficulty in passing urine for the past two years. On per abdominal examination, the uterus was enlarged to 20 weeks of gestation. Transvaginal ultrasound showed a cervical ectopic pregnancy of nine weeks and six days of gestation in the presence of a large cervical fibroid. The patient underwent emergency laparotomy with a total hysterectomy. Intraoperative findings included a highly vascular, 20-week-sized uterus and a gestational sac located in the posterior lip of the cervix. Considering rarity and possible untoward events, the case was taken under general anesthesia. Severe bleeding complicated the management, requiring internal iliac artery ligation. The patient's postoperative course was uneventful, and she was discharged on day 5.

This case report highlights the challenges in diagnosing and managing a cervical ectopic pregnancy complicated by a large cervical fibroid.

## Introduction

Cervical fibroids are rare, accounting for less than 1% of the uterine fibroids [1]. A review of literature on cervical fibroids showed that 44% present with abnormal uterine bleeding, 11% with urinary complaints, 20% with pressure symptoms, and 4% with anemia [2]. Cervical ectopic pregnancies are also rare and account for only 0.1% of all ectopic pregnancies [3]. However, it carries a high risk of severe bleeding by eroding the blood vessels and can be fatal, making them high-risk cases. Per vaginal bleeding is a common presentation and can often be painless; thus, a patient might not seek medical care early. However, uncontrolled bleeding is a factor that might require even a hysterectomy [4]. The coexistence of a cervical ectopic pregnancy with a large cervical fibroid presents a rare and challenging clinical scenario. To the best of our knowledge, such a case has not been previously reported in the literature. This case report highlights the diagnostic and management complexities encountered in such an unusual combination.

## Case presentation

**Figure 1:**
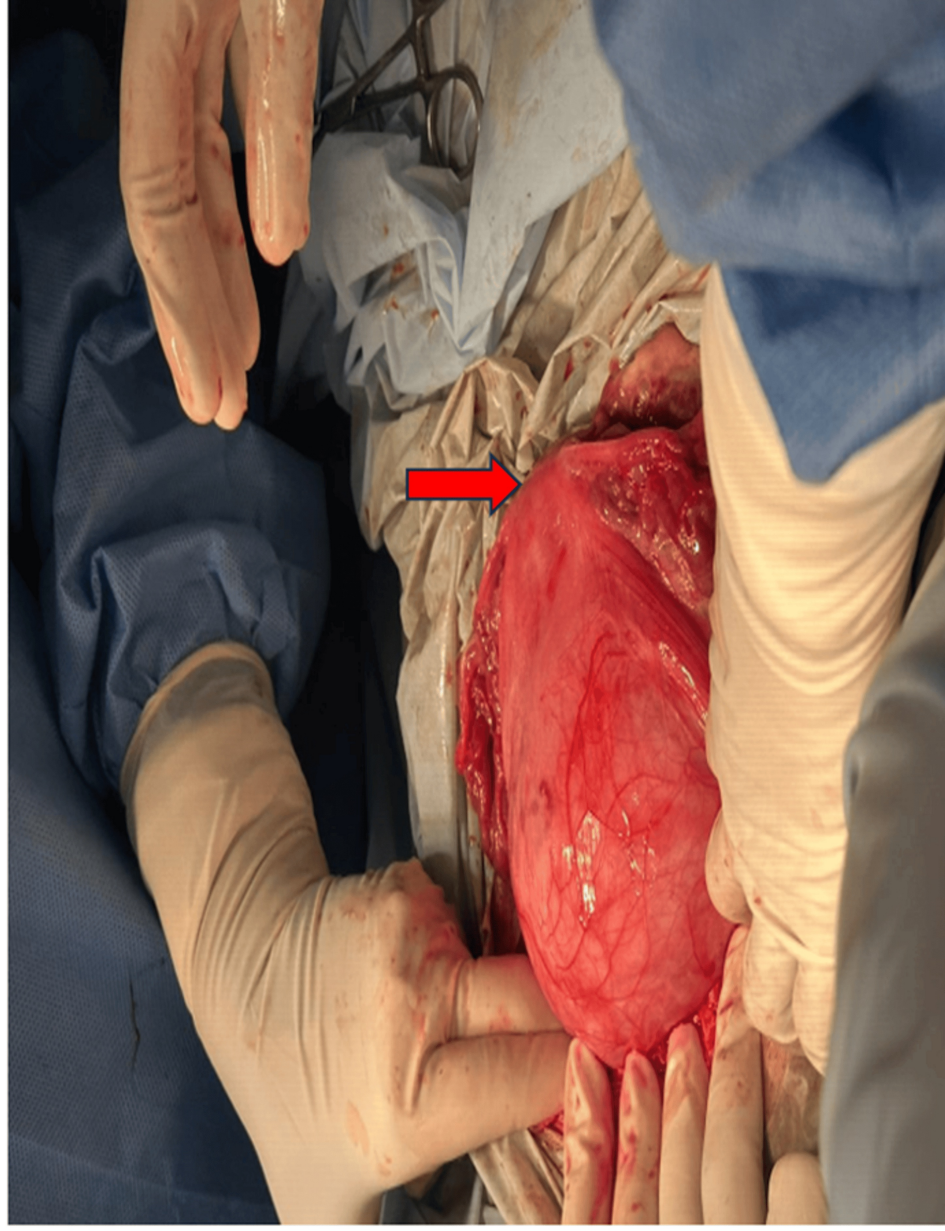
Intraoperative image demonstrating the classical “Lantern on St. Paul’s Dome” appearance of a cervical fibroid. The red arrow indicates the normal uterine fundus.

A 29-year-old woman, gravida 5 para 4 live 4, presented to our hospital emergency department with complaints of excessive bleeding for the past few hours. She previously had all institutional vaginal deliveries, which were uneventful, with her last childbirth five years back. She had a history of irregular and heavy vaginal bleeding for the past three months. She also had a history of recurrent urinary tract infections with difficulty in passing urine for the past two years. She was not on any method of contraception, with no significant medical or past history. On general examination, she appeared thin-built and pale. At admission, her pulse rate was 120/min and her blood pressure was 100/60 mmHg. On abdominal examination, a firm, mobile mass corresponding to a 20-week size was palpable. On per vaginal examination, a tense, bluish bulge was noted on the posterior lip of the cervix, while the anterior lip was thinned out and stretched over the bulge. Active bleeding was noted from the cervical os at the time of examination. The urine pregnancy card test was positive. Transabdominal ultrasound revealed a large hypoechoic lesion measuring 14 cm × 10 cm in the lower segment, suggestive of cervical fibroid. The gestational sac was not seen inside the uterine cavity. Transvaginal ultrasound showed a gestational sac with a small fetal pole and no cardiac activity, corresponding to nine weeks six days of gestation, located in the posterior lip of the cervix rather than in the cervical canal (Figure 1).

**Figure 2:**
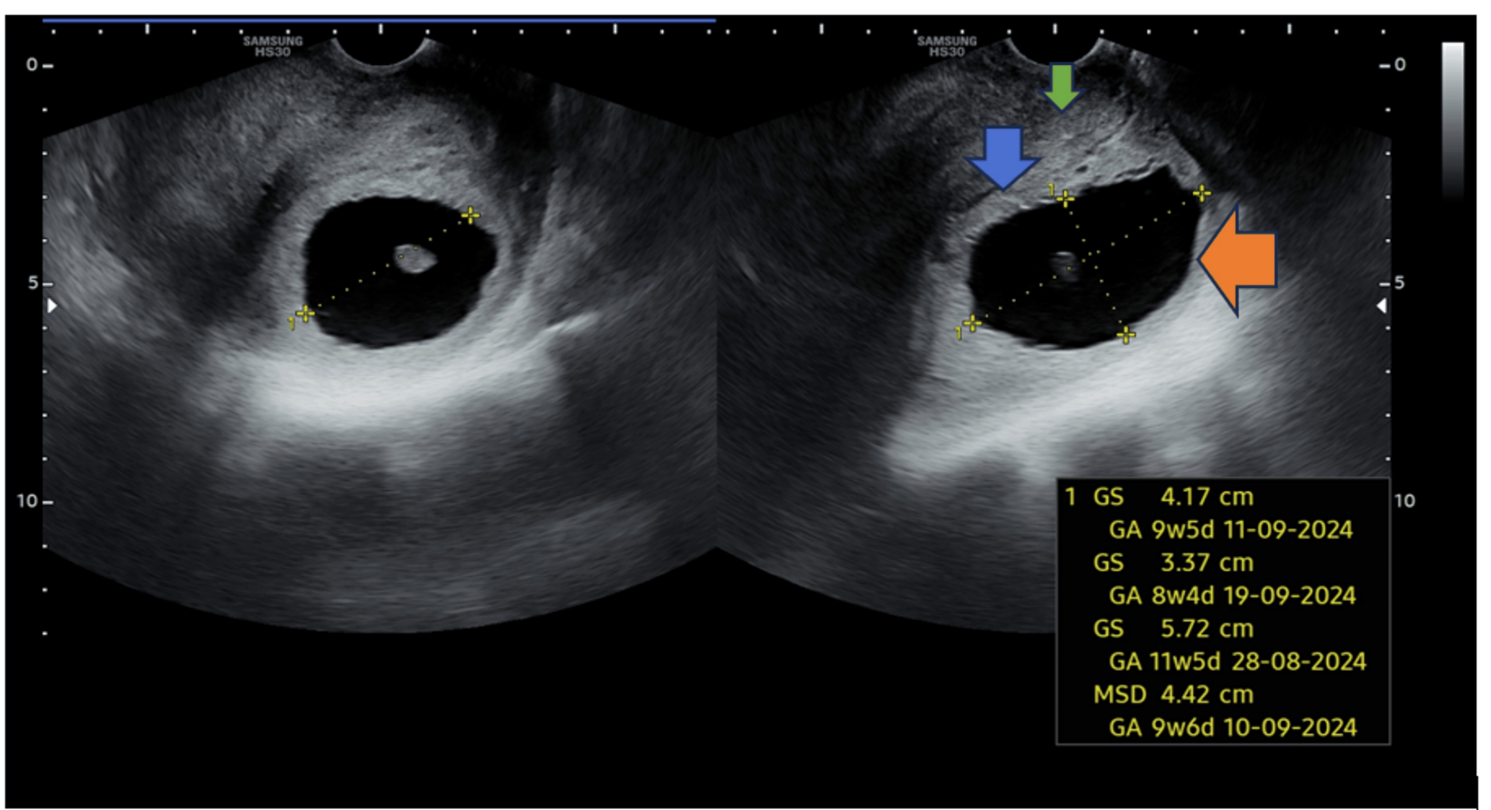
Ultrasound image showing a hypoechoic lesion representing the gestational sac (GS) with a fetal pole. The blue arrow indicates the hyperechoic line of the cervical canal; the orange arrow shows the posterior lip of the cervix with the GS; and the green arrow shows the anterior lip. GS, gestational sac; MSD, mean sac diameter; GA, gestational age

Her initial preoperative hemoglobin level was 8.4 g/dL, with otherwise normal routine blood tests. Because of active bleeding per vaginum, the patient was taken up for emergency laparotomy under general anesthesia, with written informed consent obtained, including for a possible hysterectomy. Intraoperative findings included a large, highly vascular 20-week-sized uterus with a huge cervical fibroid measuring approximately 16 × 10 cm, and a normal-sized uterus sitting on top, giving the classic Lantern on St. Paul’s Dome appearance (Figure 2). 

**Figure 3:**
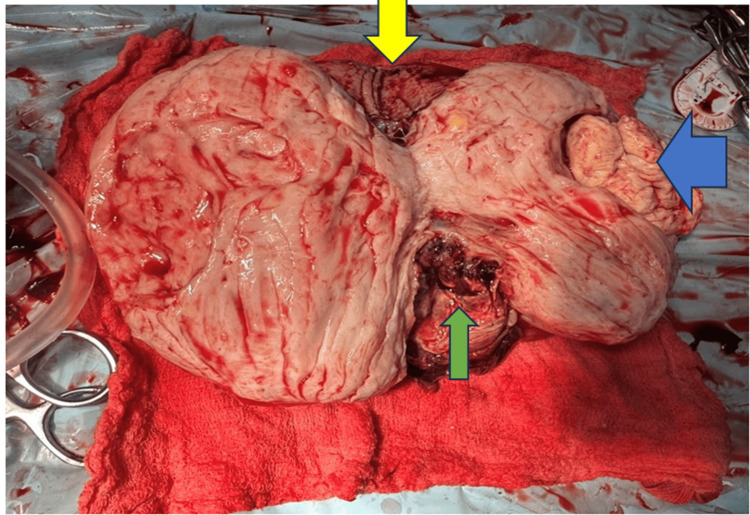
Ultrasound image showing a hypoechoic lesion representing the gestational sac (GS) with a fetal pole. The blue arrow indicates the hyperechoic line of the cervical canal; the orange arrow demonstrates the posterior lip of the cervix containing the GS; and the green arrow marks the anterior lip. GS, gestational sac; MSD, mean sac diameter; GA, gestational age

Due to the gravid state, the uterus was markedly vascular, precluding any attempt at uterine preservation via myomectomy, as this carried a significant risk of life-threatening hemorrhage. Hence, an early decision was made to proceed with a hysterectomy. Given excessive intraoperative bleeding, bilateral internal iliac artery ligation was performed to achieve hemostasis. Estimated blood loss was around 1,500 cc in the intraoperative period. Three units of packed red blood cells were transfused intraoperatively to maintain the hemoglobin and hemodynamics. The cut section of the specimen is shown in Figure 3.

Her postoperative period was uneventful, and she was discharged on day 5. Her hemoglobin level at the time of discharge was 9.3 g/dL. The histopathological report suggested a hemorrhagic mass in cervical tissue showed chorionic villi and the presence of a benign leiomyoma of the cervix. She returned for follow-up at six weeks and three months post-surgery, and her postoperative course was uneventful.

## Discussion

The present case highlights a clinical rarity, reporting a cervical ectopic pregnancy coexisting with a cervical fibroid. The presence of the fibroid not only compounded the diagnostic challenge but also influenced the management strategy, particularly in considering options for conservative management of cervical ectopic pregnancy. Cervical ectopic pregnancy is rare, and the embryo implants and develops inside the endocervical canal during cervical pregnancy. A history of dilatation and curettage, prior CS, cervical surgery, endometritis, intrauterine device use, in vitro fertilization, intrauterine myomas, uterine abnormalities, etc., are all considered to be contributing factors to cervical pregnancy [5]. Further, the present case also did not have any previous surgery, abortion, or history of assisted delivery; thus, the huge cervical fibroid could be the reason for cervical ectopic pregnancy.

The present case was also unique, considering the ectopic sac location. The usual location of cervical pregnancy is the cervical canal with a shaped cervix. However, in this case, it was unusually located in the posterior wall of the cervix. Prolonged and painless vaginal bleeding is the most common symptom of cervical pregnancy. The diagnosis is confirmed by transvaginal ultrasound, which should show a gestational sac in the cervical canal with a negative sliding organ sign [6]. The typical sign of cervical pregnancy is a bulky cervix with bluish mucosa and dilated and tortuous mucosal vessels. A lump in the abdomen is unusual in cervical pregnancy, especially without any significant risk factors, and ectopic pregnancy could have been easily missed or misdiagnosed.

The management of cervical ectopic pregnancy includes systemic methotrexate, intra-amniotic instillation of KCl and methotrexate, balloon catheter tamponade (Foley's catheter) and cerclage, operative hysteroscopy, or hysterectomy [7]. Conservative medical management is a good option and can be combined with dilatation and curettage. However, it is associated with high rates of acute bleeding complications. According to a systematic review by Nikolettos et al., total abdominal hysterectomy is the preferred management in cervical pregnancy in case of excessive bleeding, unstable vitals, pregnancy in the second trimester, associated uterine pathology, and failed medical management [8]. Literature suggests high rates of hysterectomy due to excessive bleeding during surgical management, as was done in our case. In a review, 100% of cervical pregnancies beyond 12 weeks of gestation required hysterectomy [9]. Although our patient has a gestational sac corresponding to nine weeks, because of the huge cervical fibroid, we also needed surgical management over the conservative approach.

Cervical pregnancy-related bleeding could be life-threatening and could require a hysterectomy. A scoping review of cervical ectopic pregnancy showed that the rate of hysterectomy is around 9% [10]. Furthermore, cervical fibroids - particularly when large - pose significant surgical challenges due to their proximity to vital structures such as the bladder and ureters. These cases are also associated with a high risk of intraoperative bleeding, which may necessitate hysterectomy and blood transfusion [11]. As in this case, conservative management was not attempted, given profuse and heavy bleeding from the cervical os. Intraoperative acute severe hemorrhage posed a significant challenge, necessitating bilateral internal iliac artery ligation to achieve hemostasis.

## Conclusions

Cervical ectopic pregnancy is a rare but potentially fatal illness. As in this instance, a large fibroid complicated a cervical pregnancy, increasing the risk of severe hemorrhage and the need for hysterectomy. Prompt recognition, rapid decision-making, and timely surgical intervention with hysterectomy and internal iliac artery ligation were crucial in saving the patient’s life. Early diagnosis and individualized management remain essential in reducing maternal morbidity and mortality in such rare presentations. While this case highlights hysterectomy as a definitive treatment in the setting of uncontrolled hemorrhage, it also raises important questions regarding optimal strategies for fertility preservation and minimally invasive management in more stable patients. Further studies are needed to better understand the underlying etiology, refine diagnostic approaches, and evaluate conservative management options in similar challenging cases.
